# Assessing Pain and Anxiety Impact in Smokers with Spine Fractures Managed Without Surgery: A Retrospective Cohort Study

**DOI:** 10.3390/jcm14155332

**Published:** 2025-07-28

**Authors:** Jose Castillo, James Zhou, Gabriel Urreola, Michael Nhien Le, Omar Ortuno, Matthew Kercher, Kee Kim, Richard L. Price, Allan R. Martin

**Affiliations:** Department of Neurosurgery, University of California, Sacramento, CA 95817, USA; james.zhou7143@cnsu.edu (J.Z.); miqle@ucdavis.edu (M.N.L.); oaortuno@ucdavis.edu (O.O.); mjkercher@ucdavis.edu (M.K.); kdkim@ucdavis.edu (K.K.); riprice@ucdavis.edu (R.L.P.); armartin@ucdavis.edu (A.R.M.)

**Keywords:** smoking, spine fractures, spine trauma, pain, anxiety

## Abstract

**Background/Objective:** Smoking is known to impair fracture healing and worsen surgical outcomes, but its effect on psychological recovery in spine trauma patients remains unclear. The purpose of this study is to assess how smoking affects pain and anxiety in patients with spine fractures treated either conservatively or surgically. **Methods:** We conducted a retrospective analysis looking at spine fracture patients > 18 years old seen at a single institution between 11/2015 and 9/2019. Patient variables such as age, sex, race, ethnicity, mechanism of injury, fracture location, presence of spinal cord injury, surgical intervention, hospital and ICU LOS, disposition, and EQ-5D-3L at 3 and 12 months were collected and analyzed. **Results:** Non-operative management was selected for 403 patients, of which 304 never smoked and 99 were smokers. Surgical management was utilized for 126 patients, of which 90 never smoked and 36 were smokers. Studying non-smokers and current smokers, higher levels of extreme pain and anxiety at 3 and 12 months were reported in smokers managed conservatively. Smokers managed surgically reported higher levels of pain and anxiety than non-smokers at 3 months but not at 12 months. No significant differences were seen with regards to changes in pain or anxiety between the 3- and 12-month follow-up. **Conclusions:** Smoking is independently associated with higher levels of pain and anxiety in conservatively managed spine fracture patients. These findings suggest a need for early intervention and cessation efforts in the trauma setting. Further investigation is warranted to clarify whether underlying psychological or physiological phenomena are impacting patient outcomes.

## 1. Introduction

Smoking is a large public health concern both worldwide and within the United States (US). Approximately 40 million adults report currently smoking in the US, a costly habit associated with over USD 300 billion in annual healthcare costs [1,2]. Directly responsible for the tremendous social burden are the deleterious side effects of cigarette smoke: an increased lung and breast cancer risk and increased risk of atherosclerotic heart disease and neurologic disease, amongst others [3,4,5,6]. Smoking status is particularly important to the field of surgery, where smoking has been shown to be associated with an increased risk of fractures [7], mortality, morbidity, and peri-operative complications [8,9]. This results commonly in longer hospital stays, increased rates of admission to the intensive care unit (ICU), repeat surgeries, and higher healthcare costs [10]. 

Specific to spine neurosurgery, smoking is associated with decreased fusion success rates and poor bone healing post-surgery. There is evidence that the effect of smoking on these outcomes is tied to the delayed revascularization post-op, resulting in delayed wound and bone healing [10,11,12]. However, while there are many studies analyzing the effects of smoking on physiologic surgical outcomes, such as those discussed briefly above, there are far fewer studies looking at the effects of smoking on psychological outcomes of surgical success, such as pain or anxiety. Moreover, the neurosurgical literature contains no reports that examine the short-term effects of smoking on patient pain or anxiety post-surgery for spine fractures in the trauma setting. These patients are acute cases of the surgical or conservative management of smoking patients who are treated without the opportunity for smoking cessation beforehand and represent an ideal patient population for further characterization.

This study aims to better qualify how smoking affects the pain or anxiety of spine fracture patients managed operatively or non-operatively in the trauma setting by following their progression both in the immediate and short-term post-op periods.

## 2. Methods

**Figure 1:**
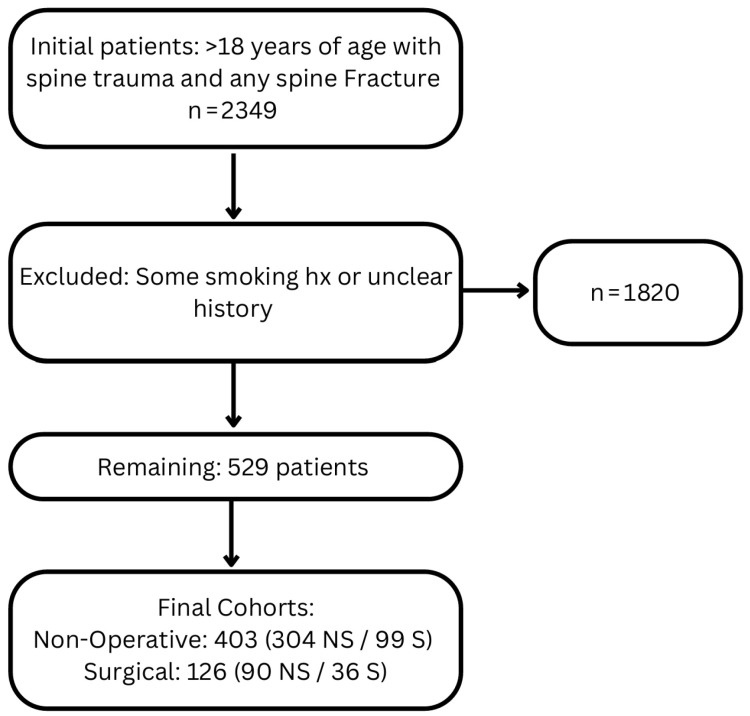
Patient Inclusion and Exclusion flowchart.

This study performed an Institutional Review Board (IRB)-exempt retrospective analysis of prospectively collected data on spine trauma patients aged over 18 years with any type of spinal fracture seen at a single institution between November 2015 and September 2019 (Figure 1). Informed consent was deemed unnecessary by the IRB due to the retrospective nature of the study. Patient variables such as age, sex, ethnicity, mechanism of injury, fracture location, presence of spinal cord injury, surgical intervention, hospital and intensive care unit (ICU) length of stay (LOS), and disposition were recorded. Quality-of-life data were gathered using the EuroQol EQ-5D-3L instrument—a standardized tool commonly used to measure health-related quality of life across five domains: mobility, self-care, usual activities, pain/discomfort, and anxiety/depression. Patients were initially divided into groups based on their surgical status, either conservatively managed patients or surgical patients. Both cohorts were then divided into groups for comparison based on their smoking status: either current smokers or never smokers. Patients with some history of smoking or unclear history were excluded. Our main variable of interest was patient pain or anxiety at 3- and 12-month follow-up, recorded from the pain or discomfort and anxiety or depression sections of the EuroQol EQ-5D-3L instrument, a validated instrument for measurement of quality of life. From the larger database, patients were also excluded if they failed to complete either 3- or 12-month follow-up appointments [13].

Categorical data was analyzed via the χ^2^ test and continuous data via the Wilcoxon rank-sum test. All statistical testing was performed with the statistical computing software R (version 4.4.1; R Core Team, 2024) [14]. The results were considered significant with *p* < 0.05 due to the exploratory nature of the analysis.

### Multivariable Analysis

We conducted multivariable linear regression analyses to examine the association between smoking status and patient-reported pain and anxiety at both 3 and 12 months following spine trauma. The primary outcomes were responses to the EQ-5D-3L pain/discomfort and anxiety/depression items, which were extracted from structured follow-up calls and recoded as ordinal scores (1 = no symptoms, 2 = moderate symptoms, and 3 = severe symptoms). Predictor variables included smoking status (current smoker vs. never smoked), age at admission, sex, race (with White as the reference), ethnicity (with non-Hispanic as the reference), BMI, and surgical treatment status.

Separate linear regression models were constructed for each outcome at each time point: 3-month pain, 3-month anxiety, 12-month pain, and 12-month anxiety. Model assumptions were assessed, and cases with missing or indeterminate responses were excluded using casewise deletion. Statistical significance was set at *p* < 0.05. All analyses were conducted using R (version 4.4.1; R Core Team, 2024) within RStudio (version 2024.12.0+467).

## 3. Results

### 3.1. Patient Demographics

An initial database search included 2349 prospectively documented patient encounters, which was narrowed down to 529 patients when applying all inclusion criteria. Patients with some history of smoking or unclear history were excluded from the study (Figure 1). Of these 529 patients, 403 were treated without surgery and 126 were treated with surgery. Within the non-surgical cohort, 304 (75.4%) and 99 (24.6%) were non-smokers and smokers, respectively (Table 1). Within the surgical cohort, 90 (71.4%) and 36 (28.6%) were non-smokers and smokers, respectively (Table 2).

In the non-surgical cohort, smokers were significantly younger (60 ± 21.0 vs. 51 ± 17.2 years, *p* < 0.001, Table 1), had a greater proportion of males (51.0% vs. 74.7%, *p* < 0.001, Table 1), had a higher prevalence of both African-American (6.5% vs. 14.1%, *p* = 0.019, Table 1) and Hispanic patients (14.5% vs. 24.4%, *p* = 0.024, Table 1), and had lower prevalence of Asian patients (11.5% vs. 3.0%, *p* = 0.012, Table 1). On the other hand, non-smokers managed conservatively were appreciated to have an increased hospital LOS (*p* < 0.05, Table 1). No other differences were found between the non-smokers and smokers regarding body mass index (BMI), mechanism of injury, spinal level injured or presence of spinal cord injury (SCI), ICU LOS, or discharge disposition.

In the surgical cohort, there were no significant differences between non-smokers and smokers regarding demographic data, mechanism of injury, injury site or spinal cord injury, hospital or ICU LOS, or discharge disposition.

### 3.2. Patient Pain and Anxiety at 3- and 12-Month Follow-Up

Non-surgical smoking patients reported significantly increased levels of extreme pain or discomfort (16.1% vs. 26.3%, *p* = 0.024, Table 3) and extreme anxiety or depression (3.9% vs. 16.2%, *p* < 0.001, Table 3) at 3-month follow-up. At 12 months, significantly fewer of these patients reported being pain-free (40.5% vs. 24.2%, *p* = 0.004, Table 3) and anxiety-free (74.7% vs. 56.6%, *p* < 0.001, Table 3). They also reported significantly higher levels of extreme pain (12.2% vs. 21.2%, *p* = 0.026, Table 3) and extreme anxiety (3.6% vs. 14.2%, *p* < 0.001, Table 3). No significant differences were seen with regards to changes in pain or anxiety between the 3- and 12-month follow-up.

Surgically managed patients that smoke reported significantly higher levels of extreme anxiety at 3-month follow-up (3.3% vs. 13.9%, *p* = 0.042, Table 4). No other significant differences were noted.

### 3.3. Multivariable Analysis

At 3 months, smoking was significantly associated with increased pain (β = 0.203, *p* = 0.003) and trended toward significance for anxiety (β = 0.119, *p* = 0.077) (Table 5). Male sex was associated with lower scores for both pain (β = −0.264, *p* < 0.001) and anxiety (β = −0.280, *p* < 0.001). African American race was significantly associated with increased anxiety (β = 0.515, *p* < 0.001) but not pain (β = 0.172, *p* = 0.131). Hispanic ethnicity was significantly associated with increased pain (β = 0.163, *p* = 0.048) but not anxiety. BMI and surgery were not significantly associated with either outcome at this timepoint.

At 12 months, smoking remained significantly associated with increased pain (β = 0.183, *p* = 0.011) and anxiety (β = 0.195, *p* = 0.003). Male sex continued to predict lower pain (β = −0.175, *p* = 0.009) and anxiety (β = −0.185, *p* = 0.002) scores. African American race was associated with increased scores for both pain (β = 0.300, *p* = 0.013) and anxiety (β = 0.367, *p* = 0.001). Hispanic ethnicity was associated with increased pain (β = 0.181, *p* = 0.040), while no significant associations were observed with anxiety. No significant associations were observed with BMI, age, or surgical status.

## 4. Discussion

This study presents a retrospective analysis examining the psychosomatic impact of smoking in adult patients with spine fractures, encompassing 529 trauma patients over a four-year period. The physiological harms of smoking, such as impaired bone healing and surgical complication, are well documented, but far less attention has been given to the effect of smoking on subjective recovery metrics. Our study uniquely investigated pain, anxiety, and depression related to spine fractures—particularly in the trauma setting. Our analysis uniquely focuses on this understudied dimension, revealing that smoking likely contributes to worse psychosocial outcomes following non-operative spine fracture management. Demographic comparisons revealed that smokers managed conservatively were more likely to be younger, male, and of African American or Hispanic background, indicating potential health disparities that warrant further investigation. These differences were not observed in the surgical cohort, and key clinical variables such as BMI, injury mechanism, injury site, and presence of spinal cord injury were largely balanced across smoking status in both groups, lending strength to the observed associations between smoking and poorer reported outcomes.

Assessing EQ-5D-3L scores for both pain and anxiety showed that for patients managed conservatively, smoking negatively impacted pain and anxiety. Smokers reported higher levels of pain and anxiety at both the 3- and 12-month follow-up, indicating that the difference may have been maintained during that time. The association remains significant after adjusting for demographic and clinical variables, suggesting an independent effect of smoking on these outcomes. Male sex was associated with significantly lower pain and anxiety at both timepoints, which may reflect sex-based differences in symptom perception, coping strategies, or treatment responses. Interestingly, African American patients reported significantly higher anxiety scores at both 3 and 12 months and higher pain at 12 months even after adjusting for other covariates. These findings suggest racial disparities in post-operative and chronic symptom burden. Hispanic ethnicity was also associated with higher pain at both timepoints, though associations with anxiety were not statistically significant. Notably, BMI and surgical status were not significantly associated with pain or anxiety at either timepoint, indicating these may play a limited role in explaining the variance in subjective outcomes in this population. These results highlight the importance of addressing modifiable risk factors such as smoking and exploring tailored psychosocial support for high-risk demographic groups.

The lack of significant differences in the rate of change of improvement or worsening pain or anxiety between non-smokers and smokers may also support a conclusion that the rate of change in these scores is similar over time for both groups. The findings for pain in non-surgical patients are consistent with previously published reports observing a significant association between smoking and pain levels, both in the general population and in spinal patients [12,15]. However, these findings, both in this study and reported, are somewhat contrasted to those published previously by Vorlat et al., who observed a non-significant difference between non-smokers and smokers managed conservatively regarding pain after thoracolumbar fracture [16]. One explanation for the differences seen by other groups might lay in the specific populations examined. Vogt et al. examined spinal patients seeing their primary physicians at one instance, and Vorlat et al. grossly examined thoracolumbar fracture patients at 12-month follow-up [12,16].

Smokers surgically reported higher levels of extreme pain at the 3-month follow-up but grossly reported similar levels of pain and anxiety as their non-smoking counterparts. These results are consistent with a recent report by Stienen et al., who observed no differences regarding pain or peri-operative complications in non-smoking or smoking patients undergoing non-instrumented lumbar spine surgery [17]. Again, it is important to emphasize the differences in patient populations, as Stienen et al. focused on patients suffering from lumbar disc herniation or lumbar disc stenosis [17]. Often seen chronically, these etiologies possess different mechanisms, presentations, and long-term prognoses than do spinal fractures in the trauma setting. It is nonetheless encouraging to observe that surgical management of both acute fracture and chronic degenerative disease results in similar pain and anxiety outcomes between non-smokers and smokers.

Looking at differences in the overall population within this study’s cohort is also critical to understanding the implications of these findings. While the surgical non-smoking and smoking cohorts showed no significant differences with regards to patient demographics, there were several differences noted in the conservatively managed patient population. These differences are important considerations when trying to evaluate the differences seen between groups. The relationship between anxiety and pain is a unique one, with anxiety having been shown to be a predictor of increased pain and decreased quality of recovery [18,19]. This is particularly relevant to this cohort, as anxiety tended to mirror pain, potentially confounding the relationship between smoking and pain or anxiety. Pain and anxiety are subjective ideas processed individually and are influenced by the social and cultural backgrounds of each patient [20,21]. While socioeconomic status is not easily defined by a single set of measurements or metrics, smoking status itself has often been associated with lower socioeconomic status, which then influences pain perception [1]. Likewise, race and ethnicity and the diverse cultural backgrounds of each patient are also closely linked with the perception of pain and anxiety disorders.

Of the many strengths of this paper, one of the most important is the patient population analyzed. By broadening our catchment to include spine fracture trauma patients managed both conservatively and surgically, a large sample size could be obtained, allowing for the testing of the relationships between smoking and pain or anxiety more concretely. Additionally, the selection of the trauma patient population itself is another strength. Trauma patients often find themselves presenting acutely to the hospital after injury. The acute decline from a potentially normal baseline health represents a larger decline than would be seen in patients with longstanding chronic disease and can be associated with higher levels of pain or anxiety. Specifically, Turk and Okifuji reported that pain brought on by a specific event (work injury, motor vehicle collision, etc.) results in increased pain severity, interference in life events, affective distress, and distracting responses [22]. By specifically examining this group of patients, characterization can begin on how a particularly vulnerable patient population experiences pain and anxiety after serious acute injury.

### Limitations

The limitations of this study include the small sample size of some groups, its retrospective design, the limited follow-up window, and it being a single-center analysis. Additionally, while this data was collected prospectively, the aim was not to characterize the effects of smoking on pain and anxiety specifically. Future studies aiming to better understand any association between the two should ideally be designed to maximize power and scope while minimizing confounding predictive variables.

Second, the reliance on self-reported data from the EQ-5D-3L instrument presents inherent limitations. While widely used, the EQ-5D-3L includes only single-item domains for pain/discomfort and anxiety/depression and thus lacks sensitivity to capture more nuanced changes in psychological health. It does not distinguish between anxiety and depression or measure objective physiological stress responses such as heart rate variability or cortisol levels. Furthermore, baseline EQ-5D-3L assessments were not routinely collected at the time of injury, preventing us from determining whether the reported psychological symptoms were pre-existing or trauma-induced.

Third, the dataset lacked key variables, such as infection rates, bone healing metrics, and detailed psychological assessments, which are required to model hypothesized mediating pathways between smoking and adverse outcomes. As a result, we could not perform a path analysis since these relationships were not directly investigated. Future studies should be prospectively designed to incorporate these variables and utilize validated instruments such as the Visual Analog Scale (VAS) or State–Trait Anxiety Inventory (STAI).

Lastly, important demographic and clinical information, such as the type and severity of injuries or detailed surgical characteristics, was not fully captured. Notably, age differed significantly between the smoking and non-smoking groups and may have confounded the outcome measures. Future research should aim to include more granular demographic and clinical data to allow for the adjustment of potential confounders and to better understand the mechanisms underlying the observed associations.

## 5. Conclusions

This study performed a retrospective analysis of 529 prospectively collected patient encounters who received either surgical or conservative management for spine fractures in the trauma setting, comparing non-smokers vs. smokers in pain and anxiety both in the immediate and short-term post-op periods. It was found that in patients managed non-operatively, smoking status is associated with higher levels of pain and anxiety. These associations were not found in patients who underwent surgical operations for their fractures. These findings help characterize a particularly vulnerable patient population, trauma patients, and potentially highlight an important relationship between current smoking and worse post-trauma outcomes.

## Figures and Tables

**Table 1:** Demographic and clinical characteristics of non-operatively managed spine fracture patients by smoking status.

	Non-Smokers (n = 304, 75.4%)	Smokers (n = 99, 24.6%)	*p*-Value
**Age (years, median ± SD)**	60 ± 21.0	51 ± 17.2	<0.001
**Sex (** **males)**	155 (51.0%)	74 (74.7%)	<0.001
**Race**			
White	237 (78.0%)	74 (74.7%)	0.508
African American	20 (6.5%)	14 (14.1%)	0.019
Asian	35 (11.5%)	3 (3.0%)	0.012
Other	11 (3.6%)	8 (8.1%)	0.097
Unreported	1 (0.3%)	0 (0.0%)	1
**Ethnicity**			
Hispanic	44 (14.5%)	24 (24.2%)	0.024
**BMI (median ± SD)**	26.6 ± 6.4	25.9 ± 4.9	0.3572
**Mechanism of injury**			
Fall	122 (40.1%)	34 (34.3%)	0.305
Motor vehicle accident	143 (47.0%)	49 (49.5%)	0.671
Other	39 (12.8%)	16 (16.2%)	0.402
**Injury spinal level**			
Fracture	246 (80.9%)	76 (76.8%)	0.370
Cervical	116 (38.2%)	31 (31.3%)	0.219
Thoracic	117 (38.5%)	44 (44.4%)	0.293
Lumbar	117 (38.5%)	38 (38.4%)	0.985
Sacral	17 (5.6%)	3 (3.0%)	0.427
Spinal cord injury	4 (1.3%)	4 (4.0%)	0.106
**Hospital LOS (days, median ± SD)**	3.07 ± 8.0	4.14 ± 11.4	0.038
**ICU LOS (days, median ± SD)**	1.69 ± 6.7	2.24 ± 6.9	0.159
**Discharge disposition**			
Home	229 (75.3%)	75 (75.8%)	0.931
Rehab	10 (3.3%)	5 (5.1%)	0.540
Skilled nursing facility	35 (1.2%)	10 (10.1%)	0.698
Outside hospital	25 (8.2%)	5 (5.1%)	0.296
Other	6 (1.6%)	4 (4.0%)	0.232

**Table 2:** Demographic and clinical characteristics of surgically managed spine fracture patients by smoking status.

	Non-Smokers (n = 90, 71.4%)	Smokers (n = 36, 28.6%)	*p*-Value
**Age (years, median ± SD)**	51.5 ± 18.7	42 ± 20.2	0.331
**Males**	60 (66.7%)	27 (75.0%)	0.361
**Race**			
White	71 (78.9%)	32 (88.9%)	0.189
African American	6 (6.7%)	4 (11.1%)	0.470
Asian	6 (6.7%)	0 (0.0%)	0.182
Other	3 (3.3%)	0 (0.0%)	0.557
Unreported	3 (3.3%)	0 (0.0%)	0.557
**Ethnicity**			
Hispanic	16 (17.8%)	4 (11.1%)	0.355
**BMI (years, median ± SD)**	27.8 ± 7.2	28.4 ± 6.7	0.972
**Mechanism of injury**			
Fall	30 (33.3%)	12 (33.3%)	1
Motor vehicle accident	48 (53.3%)	21 (58.3%)	0.611
Other	11 (13.3%)	3 (8.3%)	0.552
**Injury spinal level**			
Fracture	77 (85.6%)	32 (88.9%)	0.777
Cervical	40 (44.4%)	22 (61.1%)	0.091
Thoracic	28 (31.1%)	14 (38.9%)	0.402
Lumbar	30 (33.3%)	12 (33.3%)	1
Sacral	18 (20.0%)	5 (13.9%)	0.422
Spinal cord injury	20 (22.2%)	6 (16.7%)	0.486
**Hospital LOS (days, median ± SD)**	7.78 ± 10.7	7.26 ± 19.3	0.933
**ICU LOS (days, median ± SD)**	3.61 ± 7.0	3.68 ± 11.9	0.756
**Discharge disposition**			
Home	37 (41.1%)	16 (44.4%)	0.732
Rehab	17 (18.9%)	8 (22.2%)	0.672
Skilled nursing facility	22 (24.4%)	9 (25.0%)	0.948
Outside hospital	14 (15.6%)	2 (5.6%)	0.151
Other	0 (0.0%)	1 (2.8%)	0.286

**Table 3:** Pain and anxiety outcomes at 3 and 12 months in non-operatively managed patients.

	Non-Smokers (n = 304, 75.4%)	Smokers (n = 99, 24.6%)	*p*-Value
**Three-month follow-up**			
**Pain or discomfort**			
No pain/discomfort	77 (25.3%)	17 (17.2%)	0.096
Moderate pain/discomfort	178 (58.6%)	56 (56.6%)	0.728
Extreme pain/discomfort	49 (16.1%)	26 (26.3%)	0.024
**Anxiety or depression**			
No anxiety/depression	203 (66.8%)	59 (59.6%)	0.193
Moderate anxiety/depression	89 (29.3%)	24 (24.2%)	0.333
Extreme anxiety/depression	12 (3.9%)	16 (16.2%)	<0.001
**Twelve-month follow-up**			
**Pain or discomfort**			
No pain/discomfort	123 (40.5%)	24 (24.2%)	0.004
Moderate pain/discomfort	144 (47.4%)	54 (54.5%)	0.215
Extreme pain/discomfort	37 (12.2%)	21 (21.2%)	0.026
**Anxiety or depression**			
No anxiety/depression	227 (74.7%)	56 (56.6%)	<0.001
Moderate anxiety/depression	66 (21.7%)	29 (29.3%)	0.123
Extreme anxiety/depression	11 (3.6%)	14 (14.2%)	<0.001
**Improvement at 12 months**			
**Pain or discomfort**			
Improved pain/discomfort	94 (30.9%)	23 (23.2%)	0.143
No change	169 (55.6%)	64 (64.6%)	0.113
Worsening pain/discomfort	41 (13.5%)	12 (12.1%)	0.727
**Anxiety or depression**			
Improved anxiety/depression	59 (19.4%)	18 (18.2%)	0.788
No change	209 (68.8%)	63 (63.6%)	0.346
Worsening anxiety/depression	36 (11.8%)	18 (18.1%)	0.108

**Table 4:** Pain and anxiety outcomes at 3 and 12 months in surgically managed patients.

	Non-Smokers (n = 90, 71.4%)	Smokers (n = 36, 28.6%)	*p*-Value
**Three-month follow-up**			
**Pain or discomfort**			
No pain/discomfort	14 (15.5%)	7 (19.4%)	0.597
Moderate pain/discomfort	65 (72.2%)	22 (61.1%)	0.223
Extreme pain/discomfort	11 (12.2%)	7 (19.4%)	0.295
**Anxiety or depression**			
No anxiety/depression	59 (65.6%)	22 (61.1%)	0.638
Moderate anxiety/depression	28 (31.1%)	9 (25.0%)	0.496
Extreme anxiety/depression	3 (3.3%)	5 (13.9%)	0.042
**Twelve-month follow-up**			
**Pain or discomfort**			
No pain/discomfort	23 (25.6%)	14 (38.9%)	0.138
Moderate pain/discomfort	55 (61.1%)	16 (44.4%)	0.089
Extreme pain/discomfort	12 (13.3%)	6 (16.7%)	0.629
**Anxiety or depression**			
No anxiety/depression	65 (72.2%)	23 (63.9%)	0.357
Moderate anxiety/depression	21 (23.3%)	11 (30.6%)	0.400
Extreme anxiety/depression	4 (4.4%)	2 (5.6%)	1
**Improvement at 12 months**			
**Pain or discomfort**			
Improved pain/discomfort	25 (27.8%)	12 (33.3%)	0.536
No change	48 (53.3%)	19 (52.8%)	0.955
Worsening pain/discomfort	17 (18.9%)	5 (13.9%)	0.504
**Anxiety or depression**			
Improved anxiety/depression	16 (17.8%)	9 (25.0%)	0.358
No change	62 (68.9%)	22 (61.1%)	0.403
Worsening anxiety/depression	12 (13.3%)	5 (13.9%)	1

**Table 5:** Multivariable linear regression estimates for predictors of pain and anxiety at 3 and 12 months post-injury. Values represent unstandardized beta coefficients with standard errors and *p*-values. Reference categories used: non-smoker (smoking), female (sex), White (race), non-Hispanic (ethnicity), and no surgery (surgery).

Predictor	Pain 3 Mo (β ± SE, *p*)	Anxiety 3 Mo (β ± SE, *p*)	Pain 12 Mo (β ± SE, *p*)	Anxiety 12 Mo (β ± SE, *p*)
**Smoker**	0.20 ± 0.07, 0.003	0.12 ± 0.07, 0.077	0.18 ± 0.07, 0.011	0.20 ± 0.06, 0.0028
**Age**	0.0013 ± 0.0015, 0.38	−0.0018 ± 0.0015, 0.23	−0.0011 ± 0.0016, 0.50	−0.0009 ± 0.0015, 0.55
**Sex (Male)**	−0.26 ± 0.06, <0.001	−0.28 ± 0.06, <0.001	−0.17 ± 0.07, 0.009	−0.18 ± 0.06, 0.0023
**Race**				
African American	0.17 ± 0.11, 0.13	0.52 ± 0.11, <0.001	0.30 ± 0.12, 0.013	0.37 ± 0.11, 0.0008
Asian	−0.12 ± 0.11, 0.28	−0.10 ± 0.11, 0.37	−0.07 ± 0.12, 0.60	−0.19 ± 0.11, 0.10
Other Race	0.21 ± 0.20, 0.30	0.19 ± 0.20, 0.35	−0.01 ± 0.22, 0.96	0.12 ± 0.20, 0.56
**Ethnicity (Hispanic)**	0.16 ± 0.08, 0.048	0.12 ± 0.08, 0.14	0.18 ± 0.09, 0.040	0.04 ± 0.08, 0.60
**Surgery (Yes)**	−0.03 ± 0.07, 0.62	0.003 ± 0.07, 0.96	0.03 ± 0.07, 0.67	−0.06 ± 0.06, 0.32
**BMI**	0.0018 ± 0.0014, 0.19	0.0010 ± 0.0014, 0.47	−0.0003 ± 0.0015, 0.84	0.0013 ± 0.0013, 0.31

## Data Availability

All data is available on request. All raw data are currently stored on excel documents on a secured hospital server.
